# Impact of Time and Enzyme Concentration on Sangyod Rice Bran Hydrolysate: Phytochemicals, Antioxidants, Amino Acids, and Cytotoxicity

**DOI:** 10.1186/s12284-024-00692-1

**Published:** 2024-02-13

**Authors:** Chakkapat Aenglong, Wanwipha Woonnoi, Supita Tanasawet, Wanwimol Klaypradit, Wanida Sukketsiri

**Affiliations:** 1 Division of Health and Applied Sciences, Faculty of Science, Prince of Songkla University, Hat Yai, Songkhla, 90110 Thailand; 2 Department of Fishery Products, Faculty of Fisheries, Kasetsart University, Bangkok, 10900 Thailand; 3 Center for Advanced Studies for Agriculture and Food (CASAF), Kasetsart University, Bangkok, 10900 Thailand

**Keywords:** Rice bran, Sangyod rice, Phenolic compound, Flavonoid, Amino acids

## Abstract

This study investigated the production of Sangyod rice bran hydrolysate (SYRB) from Sangyod rice, focusing on incubation times (1, 3, and 5 h) and alcalase enzyme concentrations (0, 0.7, and 1% v/v). The results demonstrated a concentration-dependent relationship: higher alcalase concentrations increased hydrolysate yield. Prolonged incubation, especially with alcalase, enhanced substrate breakdown, further increasing hydrolysate production. The degree of hydrolysis, reflecting peptide bond cleavage, depended on both incubation time and enzyme concentration, emphasizing the role of enzyme activity in efficiency. Moreover, color analysis (L*, a*, b*) and color difference (∆E) revealed intricate changes from enzymatic hydrolysis. Proximate composition analysis showed higher protein and lipid content with increased enzyme concentration and longer incubation times, whereas ash content varied with both factors. Hydrolysate powders exhibited higher moisture content than raw rice bran, indicating the impact of the hydrolysis process. The study also explored SYRB's antioxidant properties and cytotoxicity, which were sensitive to incubation time and alcalase concentration. Longer incubation increased DPPH scavenging activity, with the highest efficacy at 3 h. Meanwhile, ABTS scavenging displayed a delicate balance with alcalase concentration. The cytotoxicity study of SYRB revealed that all concentrations of SYRB were non-toxic to C2C12 cells, with cell viability values exceeding 70%.

## Background

Rice, a staple crop globally, serves as a vital food source for a significant part of the world's population (Kumar et al. 2023). Sangyod rice (*Oryza sativa* L.) is a distinctive cultivar in the southern region of Thailand, known for its unique red pigment and cultivated in the southern regions within the Songkhla lake basin, encompassing Phatthalung, Nakhorn Si Thammarat, and Songkhla provinces (Madtohsoh et al. 2022). Additionally, Thailand annually produces approximately 28.0–30.0 million tons of Sangyod rice, valued at around 180,000–200,000 million baht, making it a primary source of income and a significant export product for the country (Rika et al. 2016). Regarding the rice milling processing, this process generates a great number of by-products, such as husk, rice bran (RB), and rice germ. RB refers to the outer layers of the grain removed during milling, historically disregarded and used as animal feed. Recent research has unveiled its untapped potential, transforming it into a valuable resource across various industries (Khongla et al. 2022). Despite the discovery of high-quality protein rich in essential amino acids, along with diverse flavonoids and phytoactive compounds in RB (Andriani et al. 2022; Ghasemzadeh et al. 2018; Zhao et al. 2018), the beneficial nutraceutical properties of hydrolysate production from Sangyod rice bran remain widely unclear and unexplored.

The extraction of bioactive components from RB has been explored using several methods such as chemical and biological method (Ahmadifard et al. 2016). The chemical methods, including acidic and alkaline treatments, are cost-effective, simple, and rapid; however, the yield of bioactive compounds from these methods is limited. Additionally, the chemical reagents used in the chemical method raise concerns regarding their toxicity. In contrast, enzymatic hydrolysis method effectively exposes and releases the highest concentration bioactive compounds without compromising nutritional value (Khongla et al. 2022). In addition, enzymatic hydrolysis is safe, more efficient, and inexpensive (Ahmadifard et al. 2016; Khongla et al. 2022). The most efficient among the enzymes employed is alcalase, as it yields RB hydrolysate with the highest protein content, protein yield, and antioxidant activity (Fathi et al. 2021). Furthermore, its extensive use in the industry for hydrolysate production underscores its efficacy (Ahmadifard et al. 2016). Many studies have emphasized the potential of RB hydrolysate as dietary or nutraceutical agents that can benefit human health. These benefits encompass antioxidant, antimicrobial, antithrombotic, antihypertensive, anti-inflammatory, and immunomodulatory effects (Andriani et al. 2022; Arun et al. 2020; Ghasemzadeh et al. 2018).

Although alcalase has been frequently used in reports for cereal protein hydrolysis, studies on the production of Sangyod rice bran, particularly considering the interplay between factors such as incubation time and alcalase concentration, are scarce. This study addressed a significant gap in scientific knowledge concerning the valuable components of Sangyod rice bran, particularly its nutritional value, antioxidative properties, and its cytotoxicity. Therefore, the objective of this study aimed to elucidate the precise conditions essential for the meticulous production of Sangyod rice bran hydrolysate (SYRB), renowned for its exceptional quality and robust bioactivity. This research delves deeply into the intricate relationship between incubation time and alcalase concentration, unraveling the subtle nuances inherent in SYRB production. Through a comprehensive analysis encompassing the amino acid profile, phytochemical constituents, in vitro antioxidant properties, and cytotoxicity, this study uncovers profound insights. Additionally, the examination of subtle yet impactful color shifts, often overlooked in previous studies, adds a layer of complexity to the research. This research not only aids in bridging existing knowledge gaps but also has the potential to enhance the quality and bioactivity of SYRBH through informed and optimized production practices.

## Materials and Methods

### Preparation of Rice Bran Hydrolysate from Sangyod Rice

The rice bran obtained from Sangyod rice (*Oryza sativa* L.) (SYRB) was obtained from a local farmer in Phatthalung province, Thailand. To prepare the samples, the SYRB underwent a drying process in a hot air oven (UN30, Memmert, Germany) at 75 °C for 24 h to reduce its moisture content. After drying, the desiccated SYRB was mixed with phosphate buffer (pH 8.0) at a ratio of 1:25 w/w, forming the control group, designated as SYRB-0A (Table 1). For the enzyme hydrolysis group, 0.7% and 1% v/v of alkaline alcalase enzyme (Reach Biotechnology, Thailand) was added to the mixture, creating SYRB-07A and SYRB-1A (Table 1). The enzymatic samples were then incubated and agitated in a water bath (WNB45, Memmert, Germany) at a constant temperature of 55 °C for 1, 3, and 5 h. After the designated incubation periods, enzymatic activity was stopped by heating the mixture to 95 °C for 15 min, followed by rapid cooling in an ice bath for 30 min. The resulting mixture was then centrifuged at 4 °C using a centrifuge (Suprema 21, Tomy, Japan) at 8000×*g* for 15 min. The supernatant was carefully separated and subjected to lyophilization to obtain a powdered form. This powdered material was stored in a polyethylene bag at −20 °C for subsequent analysis.

**Table 1:** Samples of experiment

Samples	Time (h)	Alcalase (%v/v)
SYRB	–	–
SYRB-0A-1	1	0
SYRB-07A-1	1	0.7
SYRB-1A-1	1	1
SYRB-0A-3	3	0
SYRB-07A-3	3	0.7
SYRB-1A-3	3	1
SYRB-0A-5	5	0
SYRB-07A-5	5	0.7
SYRB-1A-5	5	1

*SYRB* Sangyod rice bran hydrolysate, *0A* 0% alcalase, *07A* 0.7% alcalase, *1A* 1% alcalase

### Visual Appearance and Color

Visual documentation was carried out using a Canon 60D digital camera from Japan. Color analysis was performed using a colorimeter (UltraScan VIS, Hunter Lab Inc., USA), employing the CIELAB color system. Key parameters, including L* (lightness), a* (redness/greenness), and b* (yellowness/blueness) values, were measured and recorded. The total color difference (∆E*) was computed using Eq. (1).

1 $${\Delta E}^{*} = \sqrt{{({\Delta L}^{*})}^{2}+{({\Delta a}^{*})}^{2}+{({\Delta b}^{*})}^{2}}$$

In this equation, ∆L*, ∆a*, and ∆b* denote the differences between the sample's color parameters and those of the white standard. The white standard values are L* = 97.38, a* = -0.03, and b* = 0.17.

### Degree of Hydrolysis

The α-amino acid content was determined using a modified method from Benjakul and Morrissey (1997). Briefly, 125 μL of sample dilutions were mixed with 0.2 M phosphate buffer (2.0 mL, pH 8.2). Subsequently, a 1.0 mL solution of 0.01% 2,4,6-trinitrobenzene sulfonic acid (TNBS) was added, and the mixtures were incubated in the dark at 50 °C for 30 min. To halt the reaction, 0.1 M sodium sulfite (2.0 mL) was introduced, followed by cooling for 15 min at room temperature. The α-amino acid content was quantified at 420 nm using L-leucine as the standard (ranging from 0 to 2 mmol/L). The degree of hydrolysis (DH) was calculated using Eq. (2).

2 $${\text{DH}} (\% ) = \left( {\frac{{L_{t} - L_{0} }}{{L_{\max } - L_{0} }}} \right) \times 100$$

L_t_ represents the quantity of α-amino acid in the hydrolysate product liberated at time t, L_0_ signifies the initial amount of α-amino acid in the original SYRB, and L_max_ indicates the maximum quantity of α-amino acid obtained from SYRB after undergoing acid hydrolysis. The acid hydrolysis process involved suspending 0.1 g of SYRB in 5 mL of 6 M HCl. Sample tubes were purged with nitrogen gas and sealed tightly with screwcaps. Hydrolysis was carried out at 110 °C for 24 h. The acid-hydrolyzed sample was then filtered through a 0.22 μm syringe filter. The resulting supernatant was neutralized with 6 M NaOH before quantifying the α-amino acid content.

### Proximate Composition

Proximate composition, encompassing moisture, protein, fat, and ash content, for each sample was assessed following well-established procedures detailed in AOAC International (2000). Precisely, analytical methods No. 950.46, 920.153, 960.39, and 928.08 were utilized for moisture, protein, fat, and ash analysis, respectively. Consequently, protein extraction efficiency was calculated using Eq. (3).

3 $${\text{Protein}}\;{\text{ extraction}} \;{\text{efficiency}}\; (\% ) = \frac{{{\text{Protein}}\;{\text{ content}} \;{\text{of}} \;{\text{SYRB}} \;(\% )}}{{{\text{Total }}\;{\text{protein}}\;{\text{ content}}\;{\text{ in }}\;{\text{RB}} \;(\% )}} \times 100$$

### Amino Acid Profile

The analysis of amino acid profile followed the method outlined by Jeerakul et al. (2022). In this procedure, 0.5 g of SYRB and hydrolysate samples were hydrolyzed with 5 mL of 6 M HCl in an oil bath maintained at 110 °C for 24 h. After hydrolysis, the reaction mixture was diluted with 50 mL of high-performance liquid chromatography (HPLC) grade water and filtered through a 0.22 μm nylon membrane filter (Merck, USA) for amino acid analysis. Amino acids were detected using a fluorescence detector at 230 nm for excitation and 450 nm for emission. Identification and quantification were performed based on peak area integration, using known amounts of a mixed amino acid standard (0.2 mM solution; Agilent Technologies) for comparison. Data were expressed as grams per 100 g of protein.

### Phytochemical Content

#### Total Phenolic Content

The total phenolic content was determined using the Folin–Ciocalteu colorimetric method with a slight modification according to Sukketsiri et al. (2023). A 25 μL aliquots of extracts at a concentration of 10 mg/mL or standard solutions were mixed with 50 μL of deionized water and 50 μL of 10% Folin–Ciocalteu reagent. Neutralization was achieved by adding 100 μL of 7.5% saturated sodium carbonate solution. After incubating at room temperature for 1 h, the absorbance at 765 nm was measured using a microplate reader. A calibration curve was created using a gallic acid solution, and the total phenolic content was quantified as micrograms of gallic acid (GA) equivalent per milligram of the sample on a dry weight basis.

#### Total Flavonoid Content

Total flavonoid content was assessed using a colorimetric method with a slight modification in accordance with Sukketsiri et al. (2023). Briefly, 20 μL aliquots of extracts (10 mg/mL) or standard solutions were mixed with 120 μL of deionized water, 10 μL of 5% NaNO_2_, and 10 μL of 10% AlCl_3_·6H_2_O solution. After a 5 min incubation, 50 μL of 1 M NaOH was added. The absorbance at 510 nm was measured after a 15 min incubation, and the total flavonoid content was determined using a quercetin (QE) standard curve, expressed as micrograms of QE equivalent per milligram of dry weight.

### Fourier Transform Infrared Spectroscopy (FT-IR)

FT-IR spectra of the hydrolysate samples were analyzed with an FT-IR spectrometer (Invenio S, Bruker, USA) at room temperature. The measurement encompassed the mid-infrared (mid-IR) region, ranging from 4000 to 400 cm^−1^. Signal acquisition was automated, and spectral data analysis was conducted using the OPUS 3.0 program from Bruker.

### In Vitro Antioxidation Activity

#### DPPH Scavenging

The scavenging capability of all samples (10 mg/mL) against 2,2-diphenyl-1-picrylhydrazyl (DPPH) radicals was assessed according to the procedure described by Sukketsiri et al. (2023). The samples were mixed with 0.1 mM DPPH dissolved in methanol to maintain absorbance within a predefined range. The mixture was incubated in darkness at room temperature for 30 min. Then, the reduction in DPPH radical absorbance at 515 nm was measured using a microplate reader. Scavenging activity was quantified as the inhibition percentage using Eq. (4).

4 $${\text{DPPH}}\;{\text{ scavenging }}\;{\text{activity}} (\% ) = { }\frac{{{\text{Abs}}_{{{\text{blank}}}} - {\text{[Abs}}_{{{\text{sample}}}} - {\text{Abs}}_{{{\text{control}}}} {]}}}{{{\text{Abs}}_{{{\text{blank}}}} }} \times 100$$

Abs_blank_ represents the absorbance of the DPPH solution, while Abs_control_ and Abs_sample_ denote the absorbance of the sample at 0 and 10 mg/mL, respectively.

#### ABTS Scavenging

The 2,2′-azino-bis-3-ethylbenzothiazoline-6-sulfonic acid (ABTS) radical scavenging ability was evaluated by incubating hydrolysate samples (10 mg/mL) with ABTS free radicals for 30 min and measuring the absorbance at 734 nm (Chotphruethipong et al. 2021). The percentage of ABTS free radical scavenging was calculated using Eq. (5).

5 $${\text{ABTS}}\;{\text{ scavenging }}\;{\text{activity}} \;(\% ) \; = \frac{{{\text{Abs}}_{{{\text{blank}}}} - {\text{[Abs}}_{{{\text{sample}}}} - {\text{Abs}}_{{{\text{control}}}} {]}}}{{{\text{Abs}}_{{{\text{blank}}}} }} \times 100$$

Abs_blank_ represents the absorbance of the ABTS radical solution, while Abs_control_ and Abs_sample_ denote the absorbance of the sample at 0 and 10 mg/mL, respectively.

#### Determination of Ferric Reducing Antioxidant Power (FRAP)

In the FRAP assay, hydrolysate samples (10 mg/mL) were mixed with the FRAP reagent and incubated for 30 min. The absorbance was measured at 593 nm, following the method outlined by Chotphruethipong et al. (2021). The FRAP value of the extract was determined by referencing a calibration curve constructed using ferrous sulfate standards.

### Cytotoxicity of Hydrolysate Samples in C2C12 Cells

C2C12 cells (American Type Culture Collection) were plated at a density of 10^4^ cells per well in 96-well plates and exposed to various concentrations of hydrolysate samples for 24 h. Cell viability was determined using the 3-(4,5-dimethylthiazole-2-yl)-2,5-diphenyltetrazolium bromide (MTT) assay, and the cell viability percentage was calculated using the following Eq. (6):

6 $${\text{Cell }}\;{\text{viability}} \left( \% \right) = { }\frac{{{\text{Abs}}_{{{\text{treatment}}}} }}{{{\text{Abs}}_{{{\text{control}}}} }} \times 100$$

### Statistical Analysis

The physical and chemical analysis were presented as the mean ± standard deviation (*n* = 3). Cytotoxicity results were presented as the mean ± standard error of mean (*n* = 5). Variations among three or more groups were assessed using one-way ANOVA, with subsequent pairwise comparisons conducted using the Duncan *post-hoc* test. Statistical significance was defined as *P* ≤ 0.05.

## Results and Discussion

### General Physical and Chemical Properties

#### Product Yield

This study investigated the impact of incubation time and enzyme concentrations on the yield of SYRB powder. Table 2 presents the results for different treatments with varying incubation times (1, 3, and 5 h) and alcalase concentrations (0, 0.7, and 1% v/v). Increasing alcalase concentration from 0 to 0.7% v/v and further to 1% v/v resulted in a progressive and statistically significant increase in hydrolysate yield (*P* ≤ 0.05). These results indicated that elevated enzyme concentrations improve hydrolysis efficiency, resulting in a broader breakdown of the substrate and an increased production of hydrolysate (Zang et al. 2019). The effect of incubation time on hydrolysate yield was evaluated for various treatments. In SYRB-0A-1, without alcalase, the hydrolysate yield was 26.87 ± 2.51 g/100g (dry basis) after 1 h of incubation. Extending the incubation to 3 h in SYRB-0A-3 resulted in a slightly higher yield of 27.67 ± 2.52 g/100g. Prolonging the incubation to 5 h in SYRB-0A-5 led to a yield of 29.40 ± 3.13 g/100g. However, these increases were not statistically significant (*P* > 0.05) in the absence of alcalase. In SYRB with 0.7% v/v alcalase (SYRB-07A-1, SYRB-07A-3, SYRB-07A-5), longer incubation times significantly increased the yield. The hydrolysate yield rose from 43.60 ± 1.35 g/100g after 1 h of incubation to 45.82 ± 1.79 g/100g after 3 h (*P* ≤ 0.05) and 44.16 ± 1.82 g/100g after 5 h (*P* ≤ 0.05). A similar significant trend was observed for treatments with 1% v/v alcalase concentration (SYRB-1A-1, SYRB-1A-3, and SYRB-1A-5) (*P* ≤ 0.05). These results indicated that longer incubation times significantly affect hydrolysate yield, suggesting a more extensive hydrolysis process, leading to increased substrate conversion and higher yields of the hydrolysate product (Fathi et al. 2021).

**Table 2:** Hydrolysate product yield, degree of hydrolysate, color value, visualize appearance, proximate composition, and extraction efficiency of Sangyod rice bran hydrolysate (SYRB)

Properties	SYRB	SYRB-0A-1	SYRB-07A-1	SYRB-1A-1	SYRB-0A-3	SYRB-07A-3	SYRB-1A-3	SYRB-0A-5	SYRB-07A-5	SYRB-1A-5
Hydrolysate product yield (g/100 g, dry basis)	–	26.87 ± 2.51^c^	43.60 ± 1.35^ab^	42.82 ± 1.37^b^	27.67 ± 2.52^c^	45.82 ± 1.79^a^	44.30 ± 2.55^ab^	29.40 ± 3.13^c^	44.16 ± 1.82^ab^	46.32 ± 0.70^a^
Degree of hydrolysate (%)	–	46.15 ± 2.09^e^	55.12 ± 3.20^c^	58.72 ± 2.09^c^	57.26 ± 1.21^c^	72.94 ± 4.36^b^	76.67 ± 2.09^b^	67.52 ± 2.42^d^	84.62 ± 2.09^a^	81.20 ± 1.21^a^
Color										
L^*^	42.03 ± 0.01^ g^	64.41 ± 0.04^d^	59.48 ± 0.02^f^	61.00 ± 0.02^e^	65.98 ± 0.01^c^	65.80 ± 0.12^c^	64.80 ± 0.01^d^	69.58 ± 0.01^b^	71.14 ± 0.00^a^	68.98 ± 0.01^b^
a^*^	9.39 ± 0.01^c^	11.01 ± 0.01^a^	10.72 ± 0.01^b^	11.27 ± 0.00^a^	10.48 ± 0.01^b^	10.52 ± 0.02^b^	9.99 ± 0.01^b^	9.73 ± 0.01^bc^	9.28 ± 0.01^c^	9.45 ± 0.01^c^
b^*^	8.55 ± 0.02^e^	16.14 ± 0.01^a^	12.35 ± 0.01^d^	13.48 ± 0.02^bc^	14.14 ± 0.01^b^	16.26 ± 0.03^a^	13.04 ± 0.01^c^	12.55 ± 0.01^d^	12.79 ± 0.01^d^	13.96 ± 0.02^b^
ΔE	–	23.69 ± 0.02^e^	17.91 ± 0.01^ g^	19.69 ± 0.03^f^	24.62 ± 0.01^d^	25.01 ± 0.12^c^	23.22 ± 0.01^e^	27.84 ± 0.00^b^	29.42 ± 0.01^a^	27.49 ± 0.00^b^
Visual appearance										
Proximate composition (g/ 100 g, dry basis)										
Protein	5.12 ± 0.02^a^	1.54 ± 0.02^c^	3.72 ± 0.06^b^	3.81 ± 0.04^b^	1.44 ± 0.03^c^	3.71 ± 0.07^b^	3.84 ± 0.04^b^	1.51 ± 0.01^c^	3.81 ± 0.05^b^	3.97 ± 0.05^b^
Lipid	19.51 ± 0.18^a^	1.36 ± 0.05^c^	1.48 ± 0.04^c^	1.42 ± 0.03^c^	1.44 ± 0.08^c^	1.48 ± 0.05^c^	1.48 ± 0.04^c^	1.74 ± 0.02^b^	1.76 ± 0.04^b^	1.72 ± 0.04^b^
Ash	10.96 ± 0.07^a^	6.06 ± 0.13^bc^	4.58 ± 0.13^d^	4.32 ± 0.17^de^	6.26 ± 0.14^b^	4.25 ± 0.12^e^	4.39 ± 0.12^de^	5.82 ± 0.09^c^	4.61 ± 0.23^d^	4.41 ± 0.29^de^
Moisture^♣^	2.24 ± 0.29^b^	4.80 ± 0.18^a^	4.36 ± 0.39^a^	4.61 ± 0.42^a^	4.35 ± 0.13^a^	4.63 ± 0.20^a^	4.45 ± 0.27^a^	4.39 ± 0.33^a^	4.35 ± 0.13^a^	4.61 ± 0.30^a^
Protein extraction efficiency (%	100 ± 0.00^a^	30.10 ± 0.49^f^	70.81 ± 0.91^e^	74.40 ± 0.79^c^	28.21 ± 0.49^f^	72.51 ± 1.07^d^	75.05 ± 0.90^c^	29.58 ± 0.03^f^	74.53 ± 0.66^c^	79.61 ± 0.88^b^

Values are presented as mean ± SD (n = 3). Different lowercase letters in the same row indicates significant difference (*P* ≤ 0.05)

^♣^Data is based on wet basis. For the condition of SYRB, SYRB-0A-1, SYRB-07A-1, SYRB-1A-1, SYRB-0A-3, SYRB-07A-3, SYRB-1A-3, SYRB-0A-5, SYRB-07A-5, and SYRB-1A-5, please see in Table 1

#### Degree of Hydrolysate

The degree of hydrolysate, which assesses peptide bond cleavage in a protein hydrolysate (Fathi et al. 2021), was examined to evaluate the influence of incubation time and enzyme concentration (Table 2). SYRB-07A-5 and SYRB-1A-5 yielded the highest hydrolysate degrees (*P* ≤ 0.05), indicating that prolonged incubation and higher enzyme concentrations effectively promote extensive peptide bond breakdown. Among samples with the same incubation time, SYRB-07A-3 and SYRB-1A-3 exhibited significantly higher degrees of hydrolysate than SYRB-0A-3 (*P* ≤ 0.05), implying that adding 0.7% and 1% alcalase concentrations during shorter incubation periods led to more substantial hydrolysis and increased degrees of hydrolysate. Similarly, SYRB-07A-1 and SYRB-1A-1 showed significantly higher degrees of hydrolysate compared to SYRB-0A-1 (*P* ≤ 0.05), with SYRB-07A-1 at 55.12 ± 3.20% and SYRB-1A-1 at 58.72 ± 2.09% (Table 2). These results suggested that prolonged incubation allows for a more extensive hydrolysis, resulting in a higher yield of the desired hydrolysate. Moreover, a higher enzyme concentration enhances the hydrolysis reaction's efficiency, leading to improved hydrolysate production. In contrast, alcalase-free samples (SYRB-0A-1, SYRB-0A-3, and SYRB-0A-5) generally yielded lower degrees of hydrolysate compared to enzyme-treated samples (Table 2; *P* ≤ 0.05), underscoring the pivotal role of enzyme activity in facilitating effective peptide bond cleavage. This is consistent with previous studies Thamnarathip et al. (2016), which demonstrated a higher protein yield in alcalase-treated hydrolysates attributed to increased protein and soluble component extraction. The data revealed a positive correlation between hydrolysate yield and degree of hydrolysate, emphasizing the beneficial impact of alcalase on both parameters (Hall et al. 2017).

#### Color

We investigated the influence of incubation time and enzyme concentrations on color parameters (L*, a*, b*) and color difference (ΔE), as outlined in Table 2. Statistical analysis revealed that longer incubation times increased lightness (L*) and color differences (ΔE). Simultaneously, enzyme concentrations significantly affected lightness (L*), the red-green (a*) axis, and the yellow-blue (b*) axis (*P* ≤ 0.05). A 0.7% enzyme concentration generally increased lightness and enhanced yellow-blue perception, while a 1% enzyme concentration heightened red-green perception. Our findings emphasize the significant influence of both incubation time and enzyme concentrations on color parameters and differences (Table 2; *P* ≤ 0.05). Longer incubation times and enzyme concentrations lead to more pronounced changes in lightness and color difference, attributed to the Maillard reaction involving amino acids and reducing sugars (Arsa and Theerakulkait 2015). Color values may be linked to enzymatic hydrolysis conditions, including reaction time and the type of protease enzyme (Alahmad et al. 2023). Additionally, the combined effect of prolonged incubation and increased enzyme concentration may lead to synergistic interactions, amplifying the impact on polyphenol and flavonoid transformations and resulting in a more pronounced color shift. Prolonged incubation times and increased enzyme concentrations intensify the enzymatic hydrolysis process, leading to the degradation of polyphenols and flavonoids within SYRB. This degradation results in the breakdown of complex polyphenolic structures into simpler compounds, ultimately altering the overall composition of SYRB.

#### Proximate Composition

Table 2 displayed the proximate composition of SYRB, including protein, lipid, ash, and moisture content. SYRB had significantly higher protein content compared to all other groups (*P* ≤ 0.05). Groups with enzyme concentrations of 0.7 and 1% exhibited similar protein content (*P* > 0.05), which was significantly higher than the group without enzyme (*P* ≤ 0.05). This indicates that alcalase enzyme benefited the protein content of SYRB, resulting in higher protein content in the hydrolysate. Similar findings were reported in previous research by Thamnarathip et al. (2016), which alcalase (1%) was found to increase protein content. Consequently, the influence of incubation time and enzyme concentration on lipid, ash, and moisture content (Table 2). Both time and enzyme concentration significantly affected lipid content. Extending the incubation time from 1 to 5 h resulted in a notable increase in lipid content (*P* ≤ 0.05). Similarly, elevating the enzyme concentration also led to a significant rise in lipid content (Table 2; *P* ≤ 0.05). These results align with findings reported by Cho et al. (2013), who attributed the increase to the breakdown of cell wall structures during enzymatic hydrolysis, facilitating lipid recovery. Additionally, pH played a pivotal role in lipid extraction, with higher pH (alkaline conditions) enabling more efficient lipid extraction compared to lower pH levels (Fogang Mba et al. 2021). The control group had elevated ash content due to the addition of alkali, required for pH adjustment and control during the hydrolysis process, as highlighted by Hall et al. (2017). Our study indicated that both incubation time and enzyme concentration significantly influenced the ash content of the samples. Increased enzyme concentrations consistently led to reduced ash content, emphasizing the vital roles of these factors in determining the samples' ash content (Table 2). This change in ash content in hydrolysate products could be attributed to the enzymatic breakdown of chemical bonds between organic and inorganic components, facilitated by the protease enzyme employed in the enzymatic process, as highlighted by Alahmad et al. (2023). Moisture content analysis showed that hydrolysate powders had significantly higher moisture content compared to SYRB (Table 2; *P* ≤ 0.05). Among the hydrolysate samples, slight fluctuations in moisture content were observed with varying incubation times and enzyme concentrations, but these changes were not statistically significant (Table 2; *P* > 0.05). Notably, a direct positive correlation was observed between hydrolysate yield and protein content. Treatments resulting in higher hydrolysate yields, such as SYRB-1A-5, displayed increased protein content, while those with lower yields, like SYRB-0A-1, had relatively lower protein content (Table 2). This pattern indicated that increased hydrolysate yields generally correlated with higher protein content in the hydrolysate. Notably, the outcomes of enzymatic trials surpassed those of the control group, aligning with documented yields for various protein hydrolysates in existing literature (Hall et al. 2017; Hunsakul et al. 2022). Moreover, a positive correlation emerged between the degree of hydrolysis and protein content, consistent with findings reported for other protein hydrolysates (Watchararuji et al. 2008). Samples with higher hydrolysate degrees, such as SYRB-07A-5 and SYRB-1A-5, showed increased protein content, while those with lower hydrolysate degrees, like SYRB-0A-1 and SYRB-0A-3, had relatively lower protein content (Table 2). This intricate interplay between hydrolysate yield, degree of hydrolysis, and resulting protein content underscores the nuanced dynamics of the enzymatic hydrolysis process (Islam et al. 2022).

#### Protein Extraction Efficiency

Table 2 provided a detailed examination of protein extraction efficiencies, benchmarked against the Kjeldahl method (AOAC International 2000), which serves as the standard technique for total protein analysis. Upon closer scrutiny of the data, intriguing findings emerge concerning specific enzyme hydrolysate conditions. Among the extraction methods, SYRB extracted using the Kjeldahl method had the highest protein extraction efficiency, surpassing all other conditions and achieving complete extraction. The alkaline hydrolysate showed lower protein extraction efficiency, falling below 50% efficiency of the Kjeldahl method. SYRB-1A-5, SYRB-07A-5, and SYRB-1A-1 exhibited commendable protein extraction efficiencies, while SYRB-0A-3, SYRB-07A-3, and SYRB-07A-1 showed moderate efficiencies. SYRB-0A-5 and SYRB-0A-1 had the least favorable protein extraction efficiencies, and SYRB-0A-3 had the lowest efficiency among all conditions (Table 2). The results clearly show that all enzyme hydrolysate conditions yield significantly higher protein extraction efficiencies compared to the 70% efficiency achieved by the Kjeldahl method. An increase in enzyme concentration and extension of incubation time positively correlated with protein content in hydrolysates during the hydrolysis process (Watchararuji et al. 2008). Likewise, the study conducted by Fathi et al. (2021) indicates that prolonging the incubation time allows for a more extensive action of alcalase on the protein, resulting in heightened extraction efficiency and increased cleavage of peptide bonds (Gong et al. 2021). Consequently, these factors significantly enhance extraction efficiency (Ceylan et al. 2023). Furthermore, the relationship between the degree of hydrolysate and protein extraction efficiency is evident. Higher degrees of hydrolysate, as seen in SYRB-07A-5 and SYRB-1A-5, correspond to significantly elevated protein extraction efficiencies (84.62 ± 2.09% and 81.20 ± 1.21%, respectively). Conversely, treatments with lower degrees of hydrolysate, like SYRB-0A-1 and SYRB-0A-3, exhibit considerably lower protein extraction efficiencies (30.10 ± 0.49% and 28.21 ± 0.49%, respectively) (Table 2). This positive correlation highlighted a crucial trend: a more extensive hydrolysis process, characterized by a higher degree of hydrolysate, results in a significantly more efficient protein extraction from the source material (Islam et al. 2022). It emphasizes the importance of the degree of hydrolysis in determining the effectiveness of protein extraction and provides insights into optimizing protein yield.

### Amino Acid Profile

The study investigated the concentrations of various amino acids, including essential amino acids (EAAs) and non-essential amino acids (NEAAs), in the total amino acid composition of SYRB. The results revealed that both incubation time and enzyme concentration influence the amino acid content in the samples. Longer incubation times generally resulted in higher amino acid amounts, and this trend was consistent for both EAAs and NEAAs (Table 3). Higher enzyme concentrations were linked to elevated amino acid levels, especially EAAs (Parrado et al. 2006). The variations in amino acid quantities were more prominent with shorter incubation times. While numerous amino acids exhibited significant differences in their levels based on both incubation time and enzyme concentration, the statistical significance varied depending on the specific amino acid and experimental condition. When comparing the changes in EAAs and NEAAs relative to SYRB as the reference, a clear pattern emerges. EAAs generally increased in their amounts compared to SYRB. For example, at the 1 h incubation time, SYRB-07A-1 displayed a significant increase in EAAs, with values of 70.52 ± 0.57, in contrast to SYRB (36.28 ± 0.79) (Table 3). This trend of increased EAAs persisted across various incubation times and enzyme concentrations, highlighting the role of enzymes and longer incubation periods in boosting EAAs production (Wang et al. 1999). In contrast, NEAAs exhibited a varied pattern of change, with some samples displaying decreased NEAAs relative to SYRB, while others showed similar or slightly higher values. For instance, at the 1 h incubation time, SYRB-07A-1 had reduced NEAAs (29.48 ± 0.72) compared to SYRB (63.72 ± 0.97). However, at the 5 h incubation time, SYRB-0A-5 showed a slightly higher NEAAs value (28.23 ± 0.83) compared to SYRB (25.60 ± 0.64) (Table 3). These observations highlighted the impact of enzymatic treatment and incubation time on amino acid composition and balance. Consequently, the study underscored the significance of both incubation time and enzyme concentration in shaping amino acid content, providing valuable insights for optimizing enzymatic processes and understanding the factors influencing amino acid production. By optimizing incubation time and enzyme concentration, it is possible to achieve higher yields of amino acids, enhancing the efficiency of amino acid production processes (Thamnarathip et al. 2016). These results align with Ahmadifard et al. (2016), indicating that allowing alcalase to act for a longer duration provides more time for the enzyme to interact with the protein molecules in the hydrolysates, resulting in enhanced extraction efficiency (Hanmoungjai et al. 2002). Longer incubation times promote more significant cleavage of peptide bonds, a critical step in the hydrolysis process. This increased cleavage enhances extraction efficiency by breaking down proteins into smaller, more easily extracted peptide fragments.

**Table 3:** Amino profile of Sangyod rice bran hydrolysate (SYRB)

Amino acid	SYRB	SYRB-0A-1	SYRB-07A-1	SYRB-1A-1	SYRB-0A-3	SYRB-07A-3	SYRB-1A-3	SYRB-0A-5	SYRB-07A-5	SYRB-1A-5
Essential amino acid										
Histidine	3.09 ± 0.05^a^	0.30 ± 0.09^c^	0.80 ± 0.05^b^	0.79 ± 0.03^b^	0.27 ± 0.02^c^	0.87 ± 0.02^b^	0.86 ± 0.01^b^	0.27 ± 0.02^c^	0.87 ± 0.02^b^	0.86 ± 0.01^b^
Isoleucine	1.53 ± 0.06^a^	0.22 ± 0.13^d^	0.61 ± 0.04^c^	0.66 ± 0.01^c^	0.27 ± 0.04^d^	0.75 ± 0.05^b^	0.73 ± 0.00^b^	0.27 ± 0.04^d^	0.75 ± 0.05^b^	0.73 ± 0.00^b^
Leucine	15.97 ± 0.66^a^	1.59 ± 0.34^c^	7.21 ± 0.34^b^	7.77 ± 0.04^b^	2.23 ± 0.44^c^	7.79 ± 0.53^b^	7.62 ± 0.02^b^	2.23 ± 0.44^c^	7.79 ± 0.53^b^	7.62 ± 0.02^b^
Lysine	6.82 ± 0.41^a^	1.89 ± 0.00^c^	2.55 ± 0.20^b^	2.52 ± 0.01^b^	1.66 ± 0.00^d^	2.50 ± 0.19^b^	2.39 ± 0.01^b^	1.59 ± 0.00^d^	2.50 ± 0.19^b^	2.39 ± 0.01^b^
Methionine	1.03 ± 0.08^a^	0.35 ± 0.02^d^	0.51 ± 0.05^b^	0.51 ± 0.04^b^	0.42 ± 0.04^c^	0.53 ± 0.05^b^	0.55 ± 0.00^b^	0.42 ± 0.04^c^	0.53 ± 0.05^b^	0.55 ± 0.00^b^
Phenylalanine	2.98 ± 0.09^a^	0.36 ± 0.16^c^	1.20 ± 0.09^b^	1.28 ± 0.02^b^	0.15 ± 0.04^c^	1.30 ± 0.09^b^	1.25 ± 0.00^b^	0.15 ± 0.04^c^	1.30 ± 0.09^b^	1.25 ± 0.00^b^
Threonine	1.63 ± 0.07^a^	0.24 ± 0.09^d^	0.86 ± 0.06^c^	0.91 ± 0.05^b^	0.26 ± 0.05^d^	0.94 ± 0.06^b^	0.97 ± 0.00^b^	0.26 ± 0.05^d^	0.94 ± 0.06^b^	0.97 ± 0.00^b^
Valine	2.47 ± 0.05^a^	0.48 ± 0.27^d^	1.17 ± 0.06^b^	1.15 ± 0.03^b^	0.43 ± 0.04^d^	0.86 ± 0.03^c^	0.84 ± 0.00^c^	0.43 ± 0.04^d^	0.86 ± 0.03^c^	0.84 ± 0.00^c^
Non-essential amino acid										
Alanine	3.96 ± 0.11^a^	0.59 ± 0.05^a^	2.35 ± 0.02^d^	2.49 ± 0.03^ cd^	0.63 ± 0.12^e^	2.54 ± 0.14^c^	2.50 ± 0.01^ cd^	0.63 ± 0.12^e^	2.54 ± 0.14^ cd^	2.50 ± 0.01^e^
Arginine	8.92 ± 0.41^a^	1.06 ± 0.06^c^	2.86 ± 0.36^b^	2.73 ± 0.03^b^	1.19 ± 0.17^c^	2.87 ± 0.17^b^	2.76 ± 0.00^b^	1.19 ± 0.17^c^	2.87 ± 0.17^b^	2.76 ± 0.00^b^
Aspartate	2.13 ± 0.04^a^	0.33 ± 0.10^c^	1.04 ± 0.01^b^	1.20 ± 0.02^b^	0.33 ± 0.03^c^	1.09 ± 0.04^b^	1.22 ± 0.01^b^	0.33 ± 0.03^c^	1.09 ± 0.04^b^	1.22 ± 0.01^b^
Glutamate	5.75 ± 0.14^a^	1.50 ± 0.09^d^	3.25 ± 0.09^b^	3.29 ± 0.05^b^	1.71 ± 0.09^c^	3.49 ± 0.12^b^	3.28 ± 0.05^b^	1.71 ± 0.09^c^	3.49 ± 0.12^b^	3.28 ± 0.05^b^
Glycine	8.73 ± 0.26^a^	1.04 ± 0.02^c^	3.74 ± 0.20^b^	3.83 ± 0.06^b^	1.28 ± 0.16^c^	3.97 ± 0.16^b^	3.94 ± 0.02^b^	1.28 ± 0.16^c^	3.97 ± 0.16^b^	3.94 ± 0.02^b^
Serine	2.69 ± 0.08^a^	0.37 ± 0.06^d^	1.58 ± 0.03^b^	1.69 ± 0.02^b^	0.42 ± 0.09^c^	1.66 ± 0.10^b^	1.69 ± 0.00^b^	0.42 ± 0.09^c^	1.66 ± 0.10^b^	1.69 ± 0.00^b^
Tyrosine	1.28 ± 0.04^a^	0.36 ± 0.19^c^	0.72 ± 0.42^b^	1.18 ± 0.01^a^	0.22 ± 0.03^d^	0.34 ± 0.02^c^	0.25 ± 0.00^d^	0.22 ± 0.03^d^	0.34 ± 0.02^c^	0.25 ± 0.00^d^
Cysteine	53.52 ± 1.92^a^	6.44 ± 0.09^d^	21.24 ± 1.26^b^	21.93 ± 0.21^b^	8.09 ± 1.19^c^	23.11 ± 1.28^b^	22.28 ± 0.08^b^	8.09 ± 1.19^c^	23.11 ± 1.28^b^	22.28 ± 0.08^b^
EAA (%)	36.28 ± 0.79e	64.48 ± 0.47^d^	70.52 ± 0.57^bc^	69.94 ± 0.91^c^	74.40 ± 0.73^a^	71.45 ± 0.73^b^	71.26 ± 0.47^b^	74.63 ± 0.85^a^	71.77 ± 1.01^b^	70.21 ± 0.32^bc^
NEAA (%)	63.72 ± 0.97^a^	35.52 ± 0.22^b^	29.48 ± 0.72^ cd^	30.06 ± 1.01^c^	25.60 ± 0.64^e^	28.55 ± 0.66^d^	28.74 ± 0.55^d^	25.37 ± 0.91^e^	28.23 ± 0.83^d^	29.79 ± 0.33^c^

Values are presented as mean ± SD (n = 3). Different lowercase letters in the same row indicates significant difference (*P* ≤ 0.05). *EAA* essential amino acid, *NEAA* non-essential amino acid, *ND* not detected. For the condition of SYRB, SYRB-0A-1, SYRB-07A-1, SYRB-1A-1, SYRB-0A-3, SYRB-07A-3, SYRB-1A-3, SYRB-0A-5, SYRB-07A-5, and SYRB-1A-5, please see in Table 1

### Phytochemical Content in Sangyod Rice Bran Hydrolysate (SYRB) Products

**Fig. 1:**
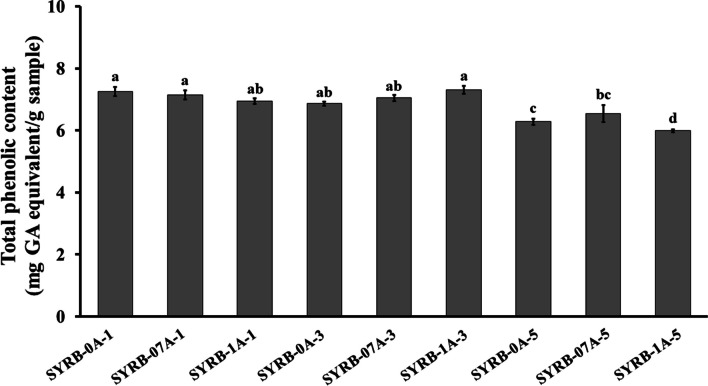
Total phenolic content in Sangyod rice bran hydrolysate (SYRB) using alcalase at various concentrations (0, 0.7, and 1% v/v) and incubation times (1, 3, and 5 h). The mean values are presented, with error bars indicating the standard error of the mean (n = 5). Statistical significance is indicated by lowercase letters (*P* ≤ 0.05). GA = gallic acid

The data analysis revealed a significant impact of incubation time on total phenolic content (Fig. 1; *P* ≤ 0.05). Comparing treatments with the same alcalase concentration, a clear decreasing trend in total phenolic content is evident as the incubation time increases from 1 to 5 h. For instance, in the SYRB-0A group, total phenolic content decreases from 7.25 ± 0.15 at 1 h to 6.28 ± 0.10 at 5 h. This decreasing trend is consistent across SYRB-07A and SYRB-1A groups (Fig. 1). These findings strongly suggested that as the incubation time is extended, there is a significant reduction in the total phenolic content across different concentrations of alcalase. Additionally, it indicates that enzyme concentrations can promote the breakdown of phenolic compounds during hydrolysis (Tian et al. 2023). Within each incubation time, increasing alcalase concentration generally decreases the total phenolic content. At a 1 h incubation time, there's a significant decrease in total phenolic content as alcalase concentration increases. SYRB-0A-1, with 0% alcalase concentration, shows higher total phenolic content compared to SYRB-07A-1 (0.7%) and SYRB-1A-1 (1%) treatment groups. This trend remains consistent at 3 h and 5 h incubation times, with total phenolic content decreasing as alcalase concentration increases (Fig. 1). These findings emphasize the critical roles of both time and alcalase concentration in determining total phenolic content. Longer incubation times tend to reduce total phenolic content, while higher alcalase concentrations generally result in lower total phenolic content within each incubation time. The reduction in phenolic content over time may result from enzymatic degradation or other chemical reactions (Tian et al. 2023). This suggested that shorter incubation times are favorable for retaining these valuable bioactive compounds. Previous research by Thamnarathip et al. (2016) observed that enzymatic hydrolysis led to little to no increase in total phenolic compounds in RB hydrolysates, indicating that extended hydrolysis might degrade phenolic compounds. A study by Puspita et al. (2017) investigated the impact of various enzymes, including alcalase, on the extraction efficiency of phenolic compounds from *Sargassum muticum*. They observed that phenolic compounds can degrade over time due to chemical reactions, enzymatic activity, and environmental factors (Puspita et al. 2017). Extended incubation times can break down phenol–protein and phenol-polysaccharide linkages, leading to phenolic compound loss (Tan et al. 2013). However, this study emphasizes the need to control incubation time for optimizing the phenolic content of RB hydrolysate, which could have applications in the food and pharmaceutical industries.

**Fig. 2:**
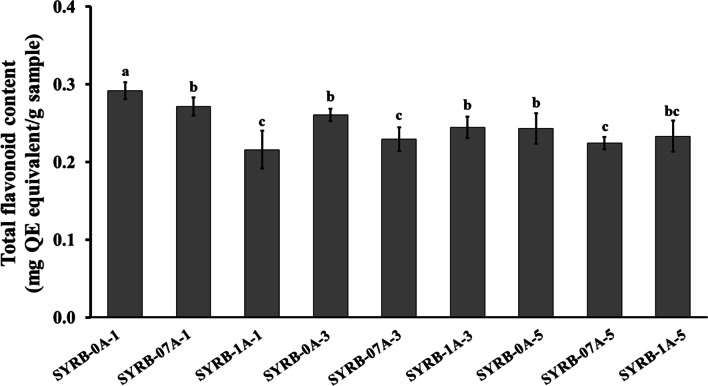
Total flavonoid content in Sangyod rice bran hydrolysate (SYRB) using alcalase at different concentrations (0, 0.7, and 1% v/v) and incubation times (1, 3, and 5 h). Mean values are shown with error bars representing the standard error of mean (n = 5). Statistical significance is denoted by lowercase letters (*P* ≤ 0.05). QE = quercetin

The total flavonoid content results, as depicted in Fig. 2, clearly highlight the impact of alcalase enzyme concentration on flavonoid content. Significant variations in total flavonoid content are observed when comparing treatments with different enzyme concentrations. Samples with 0.7% alcalase concentration (SYRB-07A-1, SYRB-07A-3, and SYRB-07A-5) generally exhibit lower total flavonoid content than those with 0% alcalase concentration (SYRB-0A-1, SYRB-0A-3, and SYRB-0A-5). Furthermore, the sample with 1% alcalase concentration (SYRB-1A-1) consistently shows the lowest total flavonoid content among all samples (Fig. 2). These observations suggested that the concentration of alcalase enzyme significantly impacts the total flavonoid content. Higher enzyme concentrations (0.7 and 1%) may lead to a decrease in the total flavonoid content compared to treatments without alcalase, a phenomenon in line with the findings of Yin et al. (2023). However, the impact of incubation time on total flavonoid content is inconclusive based on the data in Fig. 2. No consistent trend or pattern emerges in the total flavonoid content across different incubation times (1, 3, and 5 h). The total flavonoid content values vary among samples within each incubation time (Fig. 2). These findings are consistent with Yin et al. (2023), who noted that extraction time significantly influenced flavonoid yield in their study on enzymatic extraction of total flavonoids from horsetail (*Equisetum arvense*). It is important to note that prolonged incubation may lead to flavonoid degradation and oxidation, resulting in reduced flavonoid content (Zhang and Zhang 2021).

### FT-IR

**Fig. 3:**
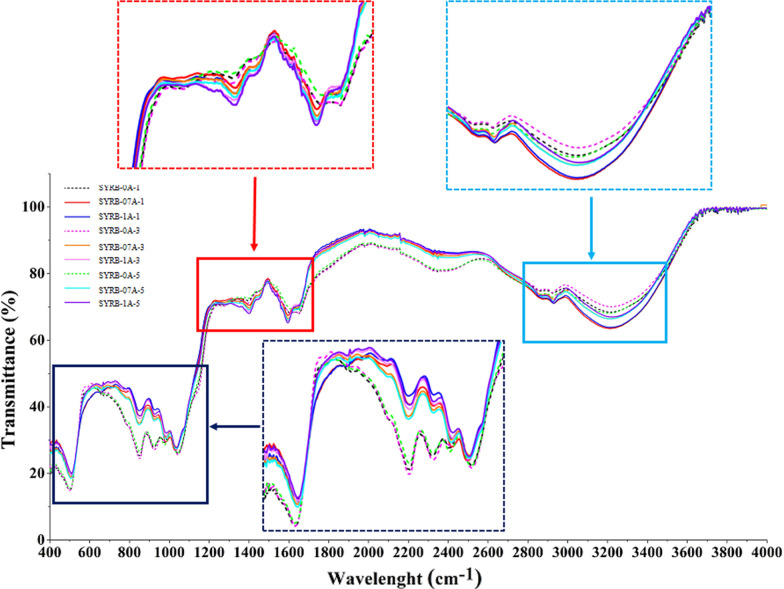
FTIR of Sangyod rice bran hydrolysate (SYRB) using alcalase at different concentrations (0, 0.7 and 1% v/v) and incubation times (1, 3, and 5 h)

FT-IR spectroscopy was used to examine the effects of incubation time (1, 3, and 5 h) and alcalase enzymatic concentration (0, 0.7 and 1% v/v) on SYRB functional groups (Fig. 3). Percent transmittance (%T) in FT-IR reveals functional group information. High %T indicates the absence or low concentration of specific groups, while low %T signifies their presence. The results indicated similar FT-IR patterns between control as alkaline-treated samples (SYRB-0A-1, 3, and 5) and enzymatic groups (SYRB-07A-1, 3, 5 and SYRB-1A-1, 3, 5) with slight shifts and %T variations (Fig. 3). The results also demonstrated a consistent trend in the FT-IR patterns across both the enzymatic groups and the control group, indicating a positive uniformity in the outcomes. The 3000–3600 cm^−1^ range correlated with OH- and NH- stretching vibrations (Tian et al. 2023). Alcalase influenced NH- stretching in the treated samples, with all alkaline-treated samples displaying lower %T. Longer incubation times had a more pronounced effect on the alkaline-treated samples, underscoring the role of incubation duration in modifying peptide bond properties, especially under alkaline conditions. Amide I (C=O stretching) occurred at 1600–1700 cm^−1^ (Daifullah et al. 2003). All alkaline-treated samples exhibited lower %T than their alcalase-treated counterparts. Extended incubation periods disrupted C=O stretching in peptide bonds, emphasizing the influence of longer reaction times on the chemical structure. Amide II (NH- bending) appeared at 1500–1600 cm^−1^ (Tian et al. 2023). Alcalase treatment resulted in lower %T values, with SYRB-1A-5 showing the lowest %T (Fig. 3). This emphasized alcalase's impact on NH- bending vibrations in peptide structures. Amide III at 1200–1300 cm^−1^ showed no significant differences between control and alcalase-treated samples (Hasanvand and Rafe 2018). Alcalase treatment led to shifts in peak positions and intensity variations, indicating transitions from β-sheet structures to α-helix or random coil formations, reflecting significant modifications in protein secondary structure (Fathi et al. 2021; Onsaard et al. 2023). Consequently, distinctive peaks associated with the C-H out-of-plane bending of aromatic compounds were evident in the 650–900 cm^−1^ range (Nguyen et al. 2022). Additionally, C–O stretching, C–C, and C=C stretching vibrations were observed between 950–1100 cm^−1^ (Nguyen et al. 2022). Moving to the aromatic ring region (1600, 1512, and 1429 cm^−1^), peaks in both samples suggested the presence of aromatic compounds such as phenols and flavonoids (Dwiwibangga et al. 2022). Notably, the FT-IR spectra illustrated that samples treated with alkaline substances exhibited lower %T values compared to those treated with alcalase. Longer incubation periods also led to lower %T values compared to shorter incubation times, which is consistent with findings from Hunsakul et al. (2021), supporting the influence of different treatments on the FTIR spectra. Consequently, the application of alcalase under different incubation durations caused significant shifts in peak positions and fluctuations in intensity within the SYRB, indicating substantial alterations in its molecular structure (Hunsakul et al. 2021).

### Antioxidation Properties

#### DPPH Scavenging

**Fig. 4:**
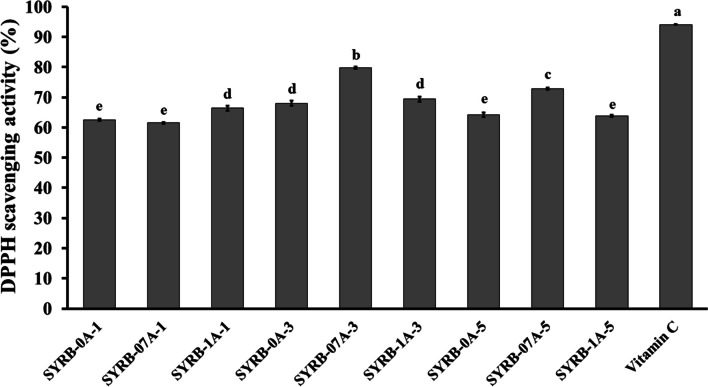
DPPH scavenging activity in Sangyod rice bran hydrolysate (SYRB) using alcalase at different concentrations (0, 0.7 and 1% v/v) and incubation times (1, 3, and 5 h). Vitamin C was used as a positive control. Mean values are represented, and error bars indicate the standard error of the mean (n = 5). Statistical significance is indicated by lowercase letters (*P* ≤ 0.05)

The analysis of DPPH scavenging activity in RB hydrolysate revealed varying antioxidant potential influenced by incubation time and alcalase concentration (Fig. 4). Longer incubation times consistently led to increased scavenging activity, with 3 h exhibiting the highest activity followed by 5 h. Alcalase concentration also played a role, with higher concentrations resulting in increased activity. Notably, 0.7% alcalase concentration generally yielded the highest scavenging activity, followed by the 1% concentration. Among the treatments, SYRB-07A-3 displayed the highest DPPH scavenging activity, effectively reducing DPPH radicals. SYRB-1A-1 also exhibited relatively high scavenging activity, while SYRB-0A-1 showed the lowest activity among the tested treatments (Fig. 4). These results can be ascribed to several factors, including the degree of hydrolysis, peptide size, and the presence of amino acids that act as electron donors, neutralizing free radicals into more stable compounds (Xiao et al. 2020). Furthermore, research by Islam et al. (2022) suggested that alcalase can target different active sites of a polypeptide, leading to an increased content of medium and low molecular weight peptides. The molecular weight distribution of proteins is closely linked to their antioxidant activity. Additionally, amino acids such as isoleucine, threonine, valine, and other hydrophobic amino acids significantly contribute to positive DPPH scavenging capability (Zhang et al. 2014). This may be attributed to the initial breakdown of peptide bonds in the hydrolysate. Park et al. (2016) reported that the effectiveness of hydrolysate's DPPH radical scavenging activity depends on the size of peptides and the type of enzyme used. However, to achieve a more comprehensive understanding of how alcalase influences antioxidant activity under consistent treatment durations, future research could delve deeper into potential interactions involving peptides, polyphenols, flavonoids, or other substances. This approach was designed to enhance clarity regarding the observed outcomes.

#### ABTS Scavenging

**Fig. 5:**
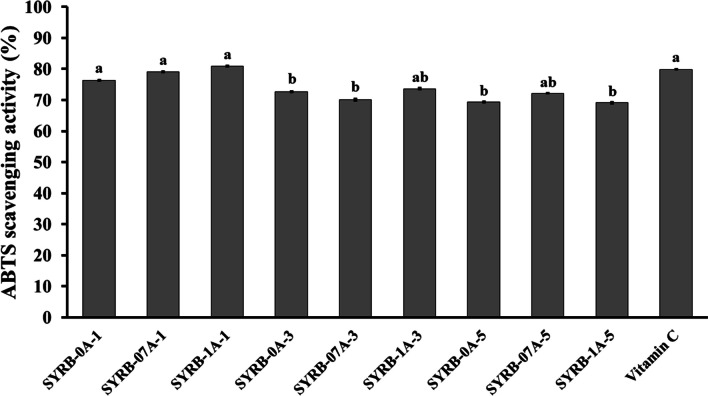
ABTS scavenging activity in Sangyod rice bran hydrolysate (SYRB) using alcalase at different concentrations (0, 0.7 and 1% v/v) and incubation times (1, 3, and 5 h). Vitamin C was used as a positive control. The mean values are presented, and the error bars indicate the standard error of the mean (n = 5). Statistical significance is indicated by lowercase letters (*P* ≤ 0.05)

The ABTS scavenging activity of rice bran hydrolysate was examined to assess its antioxidant potential (Fig. 5). The results highlighted substantial variations among the different treatment conditions, underscoring the influential roles of treatment time and alcalase concentration (*P* ≤ 0.05). When considering all treatments, it was evident that incubation time significantly impacts ABTS scavenging (*P* ≤ 0.05). For instance, at a 1 h incubation, SYRB-0A-1 displayed the highest ABTS scavenging value of 76.39 ± 0.36. However, with a 3 h incubation, ABTS scavenging values decreased across all treatments. SYRB-0A-3, SYRB-07A-3, and SYRB-1A-3 exhibited values of 72.64 ± 0.42, 70.10 ± 0.76, and 73.61 ± 0.55, respectively, with significant differences between them (*P* ≤ 0.05). Extending the incubation time to 5 h resulted in a further decline in ABTS scavenging. SYRB-0A-5, SYRB-07A-5, and SYRB-1A-5 displayed values of 69.37 ± 0.21, 72.15 ± 0.21, and 69.13 ± 0.63, respectively, with significant differences observed between SYRB-0A-1 and SYRB-0A-5, as well as SYRB-07A-5 and SYRB-0A-5 (Fig. 5; *P* ≤ 0.05). These findings suggested that extending the incubation time from 1 to 3 h and 5 h consistently reduced the ABTS scavenging activity of RB hydrolysate across all treatment conditions. This prolonged hydrolysis duration led to the degradation of peptides into free amino acids, causing a decline in ABTS activity (Saisavoey et al. 2016). Alcalase concentration significantly influenced ABTS scavenging in RB hydrolysate. SYRB-1A-1, with 1% alcalase concentration, exhibited the highest ABTS scavenging value at 80.87 ± 0.42, whereas SYRB-07A-1, with 0.7% alcalase concentration, showed a slightly lower value of 79.06 ± 0.42. Conversely, SYRB-0A-1, with 0% alcalase concentration, displayed a further reduction in ABTS scavenging with a value of 76.39 ± 0.36 (Fig. 5). These results suggested that increasing alcalase concentration from 0% to 0.7% and then to 1% progressively enhances the ABTS scavenging capacity of RB hydrolysate. This enhancement was attributed to the release of electrons and hydrogen atoms from peptides in the hydrolysate due to protein breakdown during hydrolysis, resulting in improved ABTS scavenging ability (Dey and Dora 2014). Prolonged hydrolysis might degrade peptides into free amino acids, reducing ABTS scavenging activity (Saisavoey et al. 2016). Factors such as the DH, amino acid composition within the peptide chains, and peptide molecular weight all influence the ABTS radical scavenging capability (Hunsakul et al. 2022). Additionally, future studies should consider employing a broader spectrum of enzymes or alternative hydrolysis conditions to elucidate the intricate relationships between enzymatic activity and the preservation of bioactive compounds.

#### Reducing Power

**Fig. 6:**
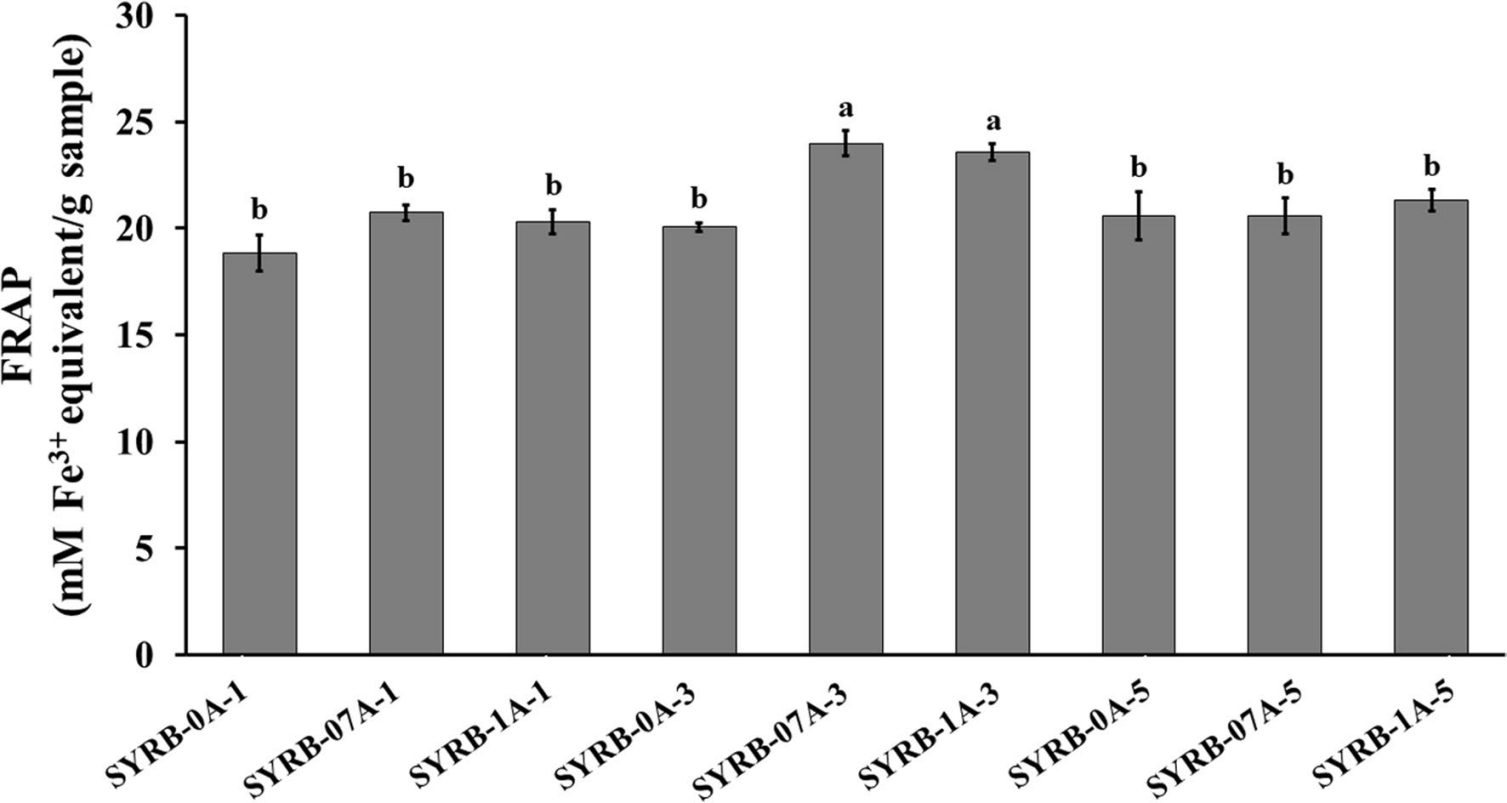
Ferric ion reducing antioxidant power (FRAP) in Sangyod rice bran hydrolysate (SYRB) using alcalase at different concentrations (0, 0.7 and 1% v/v) and incubation times (1, 3, and 5 h). The presented values are the means, and the error bars represent the standard error of the mean (n = 5). Statistical significance is indicated by lowercase letters (*P* ≤ 0.05)

The evaluation of reducing power provides key insights into the antioxidant capacity of RB hydrolysate (Fig. 6). The data revealed significant variability among different treatment conditions. This study examined the impact of incubation time on the reducing power of RB hydrolysate. After 1 h of incubation, reducing power values ranged from 18.85 ± 0.84 to 20.74 ± 0.37. Extending the incubation to 3 h resulted in values ranging from 20.06 ± 0.21 and 23.98 ± 0.61. Similarly, at 5 h, values further increased to a range of 20.59 ± 1.14 to 21.29 ± 0.50. Longer incubation periods generally led to higher reducing power in RB hydrolysate. When comparing different concentrations, a clear trend becomes evident. At 0% enzyme concentration (SYRB-0A-1, SYRB-0A-3, and SYRB-0A-5), reducing power values ranged from 18.85 ± 0.84 to 20.59 ± 1.14. At a 0.7% concentration (SYRB-07A-1, SYRB-07A-3, and SYRB-07A-5), values were higher, ranging from 20.74 ± 0.37 to 23.98 ± 0.61. With a 1% concentration (SYRB-1A-1, SYRB-1A-3, and SYRB-1A-5), values ranged from 20.29 ± 0.57 to 23.55 ± 0.40 (Fig. 6). The study demonstrated that enzyme concentration significantly boosts reducing power in RB hydrolysate, with higher concentrations resulting in increased values. Alcalase's substantial reducing power might be attributed to proton and electron generation during peptide cleavage, potentially diminishing the hydrolysate's capacity to provide reducing electrons and protons (Piotrowicz et al. 2020). Additionally, peptides containing Tyr residues are linked to enhanced antioxidant activity, indicating an improved ability to reduce or donate electrons (Cheetangdee 2014).

### Cytotoxicity

**Fig. 7:**
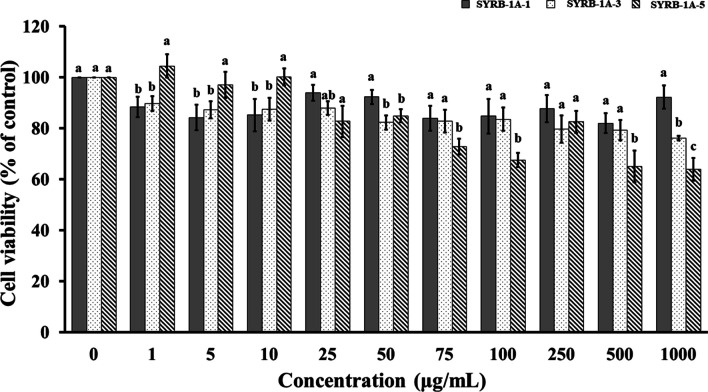
The cytotoxicity of Sangyod rice bran hydrolysate (SYRB) in C2C12 cells. The viability of C2C12 cells following a 24 h treatment with SYRB produced with alcalase at 1% v/v and various incubation times (1, 3, and 5 h) is depicted as SYRB-1A-1, SYRB-1A-3, and SYRB-1A-5, respectively. The values shown are the means, and the error bars indicate the standard error of the mean (n = 5). Statistical significance is marked with lowercase letters (*P* ≤ 0.05)

This study represented the initial exploration of the in vitro cytotoxicity of SYRB in myoblast C2C12 cells. Assessing the toxicity of natural product extracts or bioactive compounds is crucial, particularly in the context of their potential applications in human health. This raises significant safety concerns in the development of novel drugs or nutraceuticals (Morobe et al. 2012). Evaluating cytotoxicity is the primary step in the assessment of novel biological agents, and guidelines for cytotoxicity levels are typically based on their impact on in vitro cell viability percentages (López-García et al. 2014). Figure 7 presented the results of cell viability, a crucial indicator of cellular health and survival, in C2C12 cells treated with various concentrations (ranging from 0 to 1000 µg/mL) of RB hydrolysate samples (SYRB-1A-1, SYRB-1A-3, and SYRB-1A-5) over a 24 h period using the MTT assay. Remarkably, all three samples consistently maintained cell viability above 70% across the tested concentration range, meeting the acceptable threshold for cell viability. Based on previous results, SYRB-1A was chosen for the cytotoxicity test due to its superior characteristics observed in various analyses. Additionally, it displayed strong antioxidant properties, especially in the DPPH scavenging assay, highlighting its ability to neutralize free radicals. However, further investigations are necessary to comprehensively understand the underlying mechanisms and explore the potential applications of these hydrolysates in the nutraceutical or functional food context.

## Conclusions

**Fig. 8:**
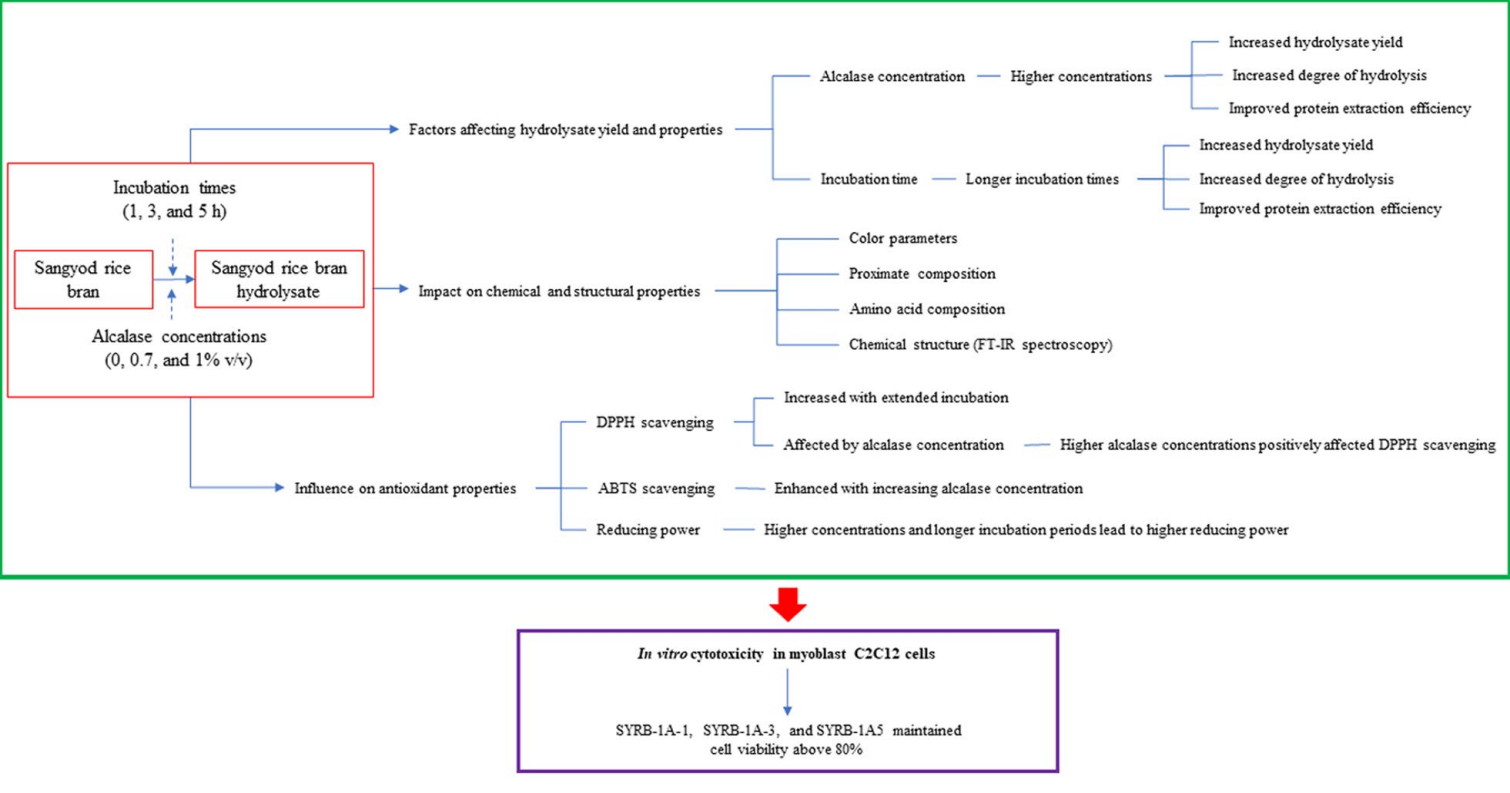
Diagram of the relationship between the incubation time and alcalase concentration on degree of hydrolysate, amino acid profile, phytochemical content, functional groups, antioxidation properties, and cytotoxicity of SYRB

Higher alcalase concentrations and longer incubation times increased hydrolysate yield, degree of hydrolysis, and protein extraction efficiency. These conditions also affected color parameters, proximate composition, amino acid composition, and SYRB's chemical structure, as determined by FT-IR spectroscopy. While longer incubation improved certain properties, it decreased phenolic content, highlighting the need for precise control over reaction duration. DPPH scavenging increased with extended incubation, enhancing SYRB's ability to reduce DPPH radicals. Higher alcalase concentrations positively affected DPPH scavenging, with SYRB-07A-3 exhibiting the highest activity. Increasing alcalase concentration from 0 to 1% enhanced ABTS scavenging, highlighting the intricate relationship between alcalase concentration and antioxidant capacity. Moreover, SYRB did not exhibit cytotoxicity, with viabilities exceeding 80% at all concentrations (Fig. 8). These findings offer valuable insights into optimizing SYRB production for applications in the food and pharmaceutical industries and underscore the need for precise control over enzymatic hydrolysis parameters to tailor SYRB properties for specific uses.

## Data Availability

The datasets used and/or analyzed during the current study are available from the corresponding author on reasonable request.
